# The Ability of Austrian Qualified Physiotherapists to Make Accurate Keep-Refer Decisions and to Detect Serious Pathologies Based on Clinical Vignettes: Protocol for a Cross-sectional Web-Based Survey

**DOI:** 10.2196/43028

**Published:** 2023-01-24

**Authors:** Wolfgang Lackenbauer, Simon Gasselich, Martina Edda Lickel, Reinhard Beikircher, Christian Keip, Florian Rausch, Manfred Wieser, James Selfe, Jessie Janssen

**Affiliations:** 1 Institute of Therapeutic and Midwifery Sciences Department of Health Sciences University of Applied Sciences Krems Krems Austria; 2 Karl Landsteiner University Krems Austria; 3 University Hospital Krems Krems Austria; 4 Department of Health Professions Manchester Metropolitan University Manchester United Kingdom

**Keywords:** red flags, clinical reasoning, physiotherapy, screening, referral, musculoskeletal

## Abstract

**Background:**

The recognition of serious pathologies affecting the musculoskeletal (MSK) system, especially in the early stage of a disease, is an important but challenging task. The prevalence of such serious pathologies is currently low. However, in our progressing aging population, it is anticipated that serious pathologies affecting the MSK system will be on the rise. Physiotherapists, as part of a wider health care team, can play a valuable role in the recognition of serious pathologies. It is at present unknown how accurately Austrian qualified physiotherapists can detect the presence of serious pathologies affecting the MSK system and therefore determine whether physiotherapy management is indicated (keep patients) or not (refer patients to a medical doctor).

**Objective:**

We will explore the current ability of Austrian qualified physiotherapists to recognize serious pathologies by using validated clinical vignettes.

**Methods:**

As part of an electronic web-based survey, these vignettes will be distributed among a convenience sample of qualified Austrian physiotherapists working in a hospital or private setting. The survey will consist of four sections: (1) demographics and general information, (2) the clinical vignettes, (3) questions concerning the clinical vignettes, and (4) self-perceived knowledge gaps and learning preferences from the perspective of study participants. Results will further be used for (1) international comparison with similar studies from the existing literature and (2) gaining insight into the participants’ self-perceived knowledge gaps and learning preferences for increasing their knowledge level about keep-refer decision-making and detecting serious pathologies.

**Results:**

Data collection took place between May 2022 and June 2022. As of June 2022, a total of 479 Austrian physiotherapists completed the survey. Data analysis has started, and we aim to publish the results in 2023.

**Conclusions:**

The results of this survey will provide insights into the ability of Austrian physiotherapists to make accurate keep-refer decisions and to recognize the presence of serious pathologies using clinical vignettes. The results of this survey are expected to serve as a basis for future training in this area.

**International Registered Report Identifier (IRRID):**

DERR1-10.2196/43028

## Introduction

We live in an aging society. The forecasted increase in the number of people older than 65 years from currently 34.4% to 59.2% by 2070 for the entire area of the European Union will pose great challenges for the health care systems of the European Union member states [1]. A very high increase in health care expenditure is to be expected [1]. In view of the challenges of an aging society, measures are being sought to cope with the increased pressure on the health care system in the future. One measure to relieve the burden on hospitals and to be able to provide the most efficient care for patients, the establishment of primary care units, is currently being propagated in Austria [2]. The core idea of primary care units is that patients are managed in a team consisting of general practitioners and different nonphysician health care professionals (such as nurses, physiotherapists, speech therapists, and occupational therapists) [2]. The expansion of primary health care in Austria is in line with the recommendations of the World Health Organization, which highlights that even though some musculoskeletal (MSK) conditions may require more specialist or surgical management, most conditions of the MSK system can readily and effectively be managed in a primary care setting [3].

MSK conditions that often present with pain and restricted mobility are highly prevalent within the general population [3]. In 2016, low back pain, for instance, was the leading cause of years lived with disability for men in 133 of 195 countries and for women in 104 of 195 countries [4].

Recent data from Denmark demonstrated that the overall prevalence of serious pathologies affecting the MSK system is at present comparatively low (2.3%) [5]. For instance, the prevalence of malignant neoplasms affecting the MSK system was 1.13% [6]. These figures are consistent with those from previous studies that reported a prevalence of metastatic spinal cancer as a serious cause of low back pain below 1% in primary care [7-10]. Another example is osteoporotic spinal fractures. The prevalence of osteoporotic fractures as a cause of vertebral pain in primary care ranges between 0.13% and 0.7% [5,9] but can be as high as 5% in a predominantly older study population [10]. In a progressively aging society, the number of people developing a serious pathology is likely to rise in the future. There is a clear link between advanced age and many cancers [11] such as prostate, lung, bowel, and breast cancer [12,13]. The significance of prostate, lung, thyroid, kidney, and breast cancer is that they account for 80% of all osseous metastases [14]. In addition, 80% of all fractures in women older than 50 years are caused by osteoporosis. One in 2 women and 1 in 5 men over the age of 50 years will experience an osteoporotic fracture [15].

The recognition of serious pathologies affecting the MSK system especially in the early stage of a disease is a challenging task [16]. However, early diagnosis is essential because this significantly improves prognosis and outcomes [16]. It is well known that especially in early stages (prodromal period), there are (if any) few or very vague/nonspecific signs and symptoms of a serious pathology [17]. However, as the disease progresses, these signs and symptoms become more obvious. Physiotherapists work very closely and sometimes over a prolonged period with their patients. They are therefore well suited to monitor the clinical situation of their patients (watchful waiting) [18] and repeatedly screen them for the occurrence or presence of specific signs and symptoms (red flags) that might indicate the presence of a serious pathology. Red flags can become apparent during the interview, physical examination, or treatment of a patient.

To analyze clinical decision-making abilities of medical and other health professionals, researchers make use of clinical vignettes that are concise paper-based or electronic descriptions of actual clinical situations [19]. Vignettes simulate real patients with various ailments and a wide range of different, sometimes complex, symptoms. Based on the clinical descriptions within such vignettes, clinicians are asked to decide about either examination procedures, (differential) diagnosis, or possible treatment options [20]. An additional advantage of vignettes is that they can be easily distributed among a large number of clinicians even with different educational backgrounds or from divergent health care settings [20,21].

Previous cross-sectional studies on qualified physiotherapists in the United States [22-26], Germany [27], Switzerland [28], Denmark [6], on Doctor of Physical Therapy students in the United States [29], and on European final year undergraduate physiotherapy students [30] have raised some concern about the physiotherapists’ ability to decide when physiotherapy is indicated (keep), or not (refer), and their ability to accurately detect the presence of serious pathologies based on clinical vignettes. Having said this, numerous case studies and case series within the literature highlight that physiotherapists can become key in the detection of serious pathologies in a clinical setting [31,32].

The ability of Austrian qualified physiotherapists to accurately detect serious pathologies and to determine if a patient is suitable for physiotherapy, or rather needs a more comprehensive medical workup, has not yet been explored. Considering the above-outlined changes to the Austrian health care sector and challenges of an increasingly aging society, this knowledge gap needs to be addressed. To fill this knowledge gap, we propose a cross-sectional web-based survey using validated clinical vignettes [6].

The overall aim of this study is to assess the ability of Austrian qualified physiotherapists to make accurate keep-refer decisions and their ability to detect serious pathologies affecting the MSK system based on 12 clinical vignettes. In addition, we want to explore potential knowledge gaps and learning preferences to acquire more expertise in making keep-refer decisions from the perspective of study participants.

## Methods

### Overview

The Methods, Results, and Discussion sections will adhere to the CHERRIES (Checklist for Reporting Results of Internet E-Surveys) statement [33].

### Ethics Considerations

The project was formally examined by the Commission for Scientific Integrity and Ethics of the Karl Landsteiner Private University, and it was found that in accordance with the set criteria, a further review by the commission was not required (project number: 1021/2022, date April 20, 2022). There were no medical ethical concerns about the conduct of the project.

### Eligibility Criteria

To be included in this study, prospective participants need to (1) be registered as qualified physiotherapists with the Austrian Health Professions Registry and (2) work in a hospital or private setting in Austria. Physiotherapists who have not actively treated patients during the last 12 months will not be eligible for this study.

### Recruitment Process

The email addresses of physiotherapists registered on the Austrian Health Professions Registry and with a publicly available email address have been collected. The recruitment process will be divided into the recruitment of physiotherapists working in a private setting and physiotherapists working in a health institution.

In the first part, physiotherapists who are working as private physiotherapists will be sent an email with an explanation of the study and a link to take part in the survey. If needed, after 2 weeks, the email will be re-sent to remind physiotherapists of the study.

In the second part, the multidisciplinary team leaders (n=44) of each health institution in Austria will be sent an email with information about the study and will be asked to provide information on the number of physiotherapists currently working for the institution and indicate if they are interested in taking part in the study. If interested, a new email will be sent to the multidisciplinary team leaders containing a link to forward to the physiotherapists to take part in the study.

When the link is activated, the physiotherapists will be taken to a website on the Unipark platform. Information regarding the research aim, eligibility criteria, and the anonymous nature of the study will be available. Consent will be given when participants accept the consent form and click on the “next” button.

### Sample Size Calculation

On March 15, 2022, a total of 16,991 physiotherapists were registered on the Austrian Health Professions Registry. Given this population, 376 physiotherapists need to be recruited to retain a 95% CI with a margin of error of 5% [34].

### Data Collection Tool

The survey will be constructed on the internet using the web-based survey tool Unipark. It will take 15 to 20 minutes to complete the whole survey. A copy of the survey, except the 12 translated vignettes, is provided in Multimedia Appendix 1.

The survey consists of four sections:

1. Demographic features of participants, for example, age, sex, working experience, and educational level.
2. Twelve vignettes, translated from English to German. After reading each clinical vignette, participants will be asked to decide if this is (a) an MSK condition, which does not need referral to a medical doctor (keep); (b) a noncritical medical condition, which needs referral to a medical doctor, while the patient can be treated by a physiotherapist (keep-refer); and (c) a critical medical condition, which needs immediate referral to a medical doctor and treatment by the physiotherapist should be stopped (refer). The vignettes (used with permission) have been validated by a panel of medical doctors and expert physiotherapists [6].
3. Feedback will be sought from participants on the 12 vignettes. Questions will be asked on
  - how difficult (on a Likert scale from 1 to 5) it is to make keep-refer decisions based on the information presented in the vignettes,
  - the relevance of the vignettes for everyday work (on a Likert scale from 1 to 5), and
  - possible missing information within the vignettes for making accurate keep-refer decisions (open-ended question).
4. Exploration of knowledge gaps and learning preferences to acquire more expertise in making keep-refer decisions and detecting serious pathologies from the perspective of study participants. Questions will be posed on
  - the confidence of participants to conduct clinical examination procedures (such as cranial nerve testing, reflex testing, and auscultation of blood vessels)
  - if participants are interested in learning more about keep-refer decision-making
  - suggestions for content and format of future training sessions to acquire more expertise in keep-refer decision-making (such as learning medium and environment/setting, teaching contents, and educational strategies)
  - any further suggestions and comments (open-ended question).

**Pilot Testing**

The first draft of this survey was sent to 5 physiotherapists and 4 medical doctors. Participants were asked to comment on their general understanding of the questionnaire and the appropriateness, comprehensibility, and proper sequencing of individual questions. In addition, the participants in the pilot could suggest additional questions that they deemed valuable. For an example, one participant suggested adding a question where future participants will be asked about their confidence in conducting clinical examination procedures (eg, cranial nerve testing) that might be relevant for clinical and keep-refer decision-making. Another suggestion was to add a question that could aid with the inclusion and exclusion criteria. Participants were therefore, right at the beginning of the survey, asked if they have treated patients during the last 12 months. If the participants answer this question with “no,” they will be thanked for their interest and then will automatically be prevented from continuing with the survey. The validity of some of the vignettes in the Austrian health care system was also questioned; therefore, after elaborate discussions, the originally envisioned vignettes by Jette et al [23] were replaced by the more current vignettes of Budtz et al [6]. The feedback from the pilot was collected and incorporated into the existing survey.

### Data Management and Analysis

The survey will be constructed online, and progression through the 12 vignettes will be mandatory so that an answer must be entered before one being able to continue. This will allow only completed clinical vignettes to be included in the analysis. Completed surveys will be automatically sent back to the online survey tool Unipark. The data will be stored at the QuestBack server park in Bremen, Germany. After the data collection is completed, the raw quantitative data will then be downloaded onto an SPSS file (IBM SPSS Statistics 28.0). The qualitative data will be exported to a Word document and then imported to a MAXQDA project (MAXQDA 2020). All data will be stored on a secure OneDrive folder to which only the researchers have access through their password protected laptops. The survey will be designed to be completely anonymous; however, there is a small risk that participants will share personal data in the open-ended question in the last section of the survey. If this happens, these data will be anonymized by a member of the research team.

Individual participants’ responses for each of the 12 vignettes will be classified as either being correct (Yes) or incorrect (No). Replicating previously used methodology [6,23,25,27-30], definitions of correct answers are as follows:

- For the MSK vignettes: treat the patient without the need for medical referral (keep) or treat the patient with additional medical checkup (keep and refer, also called watchful waiting).
- For the medical noncritical vignettes: choose to start physiotherapy with additional medical evaluation (keep and refer) or refer the patient without physiotherapy management (refer).
- The sole correct answer for medical critical vignettes is sent the patient for medical evaluation without physiotherapy management (refer).

Once the data collection period is finished, the quantitative data will be checked for completeness and outliers. Missing data will be labeled. The Shapiro-Wilk test will be used to examine if the data are normally distributed. If values are normally distributed, the mean (with SD) will be calculated; if not, the median (with IQR) will be calculated. All descriptive statistics, such as age, sex, years of experience as a physiotherapist, and type of expertise, will be collected as categorical data, and the percentage for each category will be calculated.

In the clinical vignettes, the mean percentages (plus SD) of correct keep-refer decisions and actual numbers as well as percentages of participating physiotherapists who manage to accurately answer all vignettes from a specific category will be calculated (MSK vs medical noncritical vs medical critical) [6,23,25,27-30]. If it is not normally distributed, the median percentages with IQR will be presented.

The questions concerning the feedback from study participants on the 12 vignettes, such as “how sure the participants were when answering the clinical vignettes?” and “how relevant the vignettes were for the participants?” will be calculated as percentages for each category.

The questions concerning learning about red flags, such as interest for red flags training, medium of training, and interest of specific assessment techniques, will also be listed as percentages for each category.

The 2 open-ended questions concerning missing information in the clinical vignettes and any other remarks will be analyzed deductively using framework analysis [35] in MAXQDA. The predefined codes were in line with the diagnostic physiotherapeutic process [36]: referral, medical history, and physical examination. Recurrent remarks on additional information, which could help the participants with their clinical decision-making, will be coded and combined into categories to inform the next phase of the study: the development of educational clinical case studies for physiotherapists.

## Results

The distribution of the survey took place between May 2022 and June 2022. As of June 2022, a total of 479 Austrian physiotherapists completed the survey. It is anticipated that data collection, analysis, and writing up for a potential publication in a peer-reviewed journal will take 4 to 6 months.

## Discussion

### Study Strengths

A major strength is that our methodology is in line with previously published studies [6,23,25,27-30]. In addition, we use validated clinical vignettes (with permission) that have already been used in previous research [6,23,25,27-30]. This will give us the opportunity for international comparison.

To the best of our knowledge, this is the first study that examines the ability of Austrian physiotherapists to make accurate keep-refer decisions and to recognize the presence of serious pathologies using clinical vignettes. Moreover, this is the first attempt to explore potential knowledge gaps and learning preferences to acquire more expertise in making keep-refer decisions and recognizing the presence of serious pathologies from the perspective of Austrian qualified physiotherapists. These results will be used to inform future training in this field and should help advance patient safety and interdisciplinary collaboration within the Austrian health care system.

### Study Limitations

During the preparation of the study the aim was to collect the email addresses of all physiotherapists currently registered to work in Austria. However, from the private physiotherapists not all email addresses were retrievable on the internet, and 50% of the population will not be contacted. This could have implications on the generalizability of the study.

The limitations of clinical vignettes as a sole instrument for examining clinical decision-making strategies of health care professionals have previously been highlighted [37-39]. However, vignettes have the advantage that they can be distributed to a large pool of potential participants relatively quickly and at low cost. Our approach is in line with previous research using similar methodology [6,22,23,25,27-30]. The participants in this study will have the opportunity to provide feedback on the vignettes, which will be used to develop vignettes validated for the Austrian context.

## Appendix

Multimedia Appendix 1 Red Flags survey.

Multimedia Appendix 2 External peer-review from Life Science Call 2020 - Gesellschaft für Forschungsförderung Niederösterreich m.b.H. (GFF).

## Abbreviations

CHERRIES: Checklist for Reporting Results of Internet E-Surveys
MSK: musculoskeletal

## Data Availability

We are aiming to deposit the quantitative data in a GitHub repository.
