# Supplementary material for: The Ability of Austrian Qualified Physiotherapists to Make Accurate Keep-Refer Decisions and to Detect Serious Pathologies Based on Clinical Vignettes: Protocol for a Cross-sectional Web-Based Survey
Source: JMIR Res Protoc. 2023 Jan 24;12:e43028. doi: 10.2196/43028 (PMC9906318; doi:10.2196/43028)
Supplement: Multimedia Appendix 1 [file resprot_v12i1e43028_app1.pdf]

Red Flags survey

Dear colleagues in physiotherapy,

in the context of a research project on the topic of "Red Flag Screening", the [name local university] is asking for your expertise. Specifically, the aim is to identify serious pathologies for which musculoskeletal physiotherapy is contraindicated. Your participation can contribute to the current state of knowledge of Austrian physiotherapists on the topic of Red Flag Screening. In addition, the results of the study should provide information on how future training and further education on the topic of Red Flag Screening can be designed.

You can participate in the survey if you have worked as a physiotherapist with patients in the last 12 months. Completing the questionnaire will take approximately 15-20 minutes of your time. Your participation is completely voluntary. You will not gain any direct advantage by supporting this research project or will not be disadvantaged by refusing to participate.

The end date for participation in the study is 31st of May 2022.

In this research project, we want to record whether you, as physiotherapists, can identify which patients are appropriate for physiotherapy or if medical examination and treatment is required based on 12 validated clinical vignettes.

In the second part of the survey, we want to find out how or if you as a physiotherapist would like to acquire more in-depth knowledge regarding Red Flag Screening. This information will then be used to help us develop new vignettes and plan future education within the field of Red Flag Screening.

The survey cannot be interrupted without losing the answers already entered. You can also not log into the survey for a second time from the same computer/tablet/phone, in order to ensure that the survey is not completed multiple times by the same person. Therefore, we ask that you complete the survey in one unit.

If, during the course of the survey, you wish to withdraw your participation and drop out of the study without incurring any disadvantages, simply close the digital questionnaire. The data you have already entered, however, will continue to be used.

Data collection without personal reference (anonymous data collection)

The questionnaire is designed to be anonymous, i.e. it is not possible to draw any conclusions about the person who filled in the questionnaire. Data is collected on your age (age group), gender, the period of time you have worked as a physiotherapist, level of education, and professional relationship. The core of the survey is both the response to the case studies, and the information about possible continuing education opportunities in the area of Red Flag Screening. Since this variant (without disclosure of names and contact details) lacks a personal reference in its entirety, the GDPR does not apply to the survey. The project management and project staff have access to the anonymized data. A transfer of the data, in particular to the sponsor and its contractual partners or for the purpose of any publications, will only take place in the present anonymized form.

Thank you in advance!

If you have any further questions in connection with this study, please do not hesitate to contact the project management.

Name

Name University of applied Sciences

E-mailaddress

Informed Consent

Please confirm the consent form only:

- if you fully understand the nature and procedure of the study,

- if you are willing to consent to participate, and

- if you are aware of your rights as a participant in this study.

Eligibility

The survey is aimed at physiotherapists who are currently employed.

Question 1: Have you been in direct contact with patients as a physical therapist in the last 12 months?	
Yes, I have worked with patients in the last 12 months.	No, I have NOT had direct contact with patients in the last 12 months.

Demographics

Question 1: How old are you?						
20-25	26-30	31-35	36-40	41-50	51-60	> 60
Question 2: Gender?						
Male	Female	Other	No information			
Question 3: How long have you been working as a physiotherapist?						
1 year	2-3 years	6-10 years	11-15 years	16-20 years	> 20 years	
Question 4: In which area of expertise do you predominantly work?						
Musculoskeletal	Neurology	Pediatrics	Geriatrics			
Psychiatry	Cardiorespiratory	Gynecology	Urology			
Question 5: Have you attended at least one training session focused on Red Flags Screening?						
Yes	No	No information				
Question 6: Do you practice your profession in private practice or as an employee?						
Private practice	Employed	Both				
Question 7: What is your highest degree completed in the field of physiotherapy?						
Doctorate/ PhD	Master	Bachelor	Diploma	No information		

Explanation for the vignettes

In the following section, 12 short vignettes are presented. Due to time constraints, only the most essential patient information is given, which should be used as a basis for decision-making. You have a maximum of 15 minutes to answer the 12 vignettes.

The following 3 answer options are available:

- Physiotherapy treatment
- Physiotherapy treatment and additional referral to a physician
- No physiotherapy treatment, direct referral to a physician

Only 1 answer can be selected at a time.

Clicking on "Next" starts the 15-minute time limit and the vignettes can be worked through in sequence. The questions must be answered in the order in which they occur; no question can be asked more than once.

[vignettes will be presented here]

Questions related to the 12 vignettes

Thank you for answering the vignettes!

<table>
<tr><td colspan="5">Question 1: How confident did you feel in answering the vignettes?</td></tr>
<tr><td>Highly confident.</td><td>Confident.</td><td>Less Confident.</td><td>Uncertain.</td><td>No Information.</td></tr>
<tr><td colspan="5">Question 2: How relevant are these vignettes to your everyday work?</td></tr>
<tr><td>Highly relevant</td><td>Relevant.</td><td>Little relevant.</td><td>Uncertain.</td><td>No Information.</td></tr>
<tr><td colspan="5">Question 3: What, for you, relevant information was missing in the vignettes to make a clinical decision?</td></tr>
<tr><td colspan="5"></td></tr>
</table>

Educational Tool

Question 1: Do you have the impression that the topic of "Red Flag Screening" has been sufficiently covered for you during your undergraduate training or as part of specialised postgraduate training?		
Yes	No	No information

Question 2: Are you interested in further training in the field of "Red Flag Screening"?		
Yes	No	No information

Question 3: In which setting or with which medium can you imagine to further educate yourself in this regard? (multiple answers possible)			
One day workshop in presence/attendance	Workshop lasting several days in presence/attendance	Online seminars	Hybrid Workshop (online + presence/attendance)
App	Literature (books + papers)	Websites	Other Suggestions:

Question 4: How should this continuing education be designed in concrete terms? (multiple answers possible)					
Paper based cases	Video based cases	Literature on the most current evidence	Discussion	Particle training of assessments	Other Suggestions:

Question 5: Which of the following examination procedures are you familiar with and do you use when needed? (multiple answers possible)				
Auscultation of the heart	Schellong Test 1 + 2 (Circulatory function - Orthostatic response)	Cranial nerve examination	Neurological examination of the extremities	Babinski and Klonus
Auscultation of blood vessels	Palpation of the peripheral pulses	Hoffman's Test	Inverted supinator sign	Blood pressure examination

Question 6: Feel free to leave additional comments in the text box below

Thank you for your participation!

If you are interested in knowing the answers to the vignettes, please contact us by mail [Email address].

After the end of the survey (June 2022), we will gladly send you the answers.

The survey is closed.
