# Supplementary material for: The Ability of Austrian Qualified Physiotherapists to Make Accurate Keep-Refer Decisions and to Detect Serious Pathologies Based on Clinical Vignettes: Protocol for a Cross-sectional Web-Based Survey
Source: JMIR Res Protoc. 2023 Jan 24;12:e43028. doi: 10.2196/43028 (PMC9906318; doi:10.2196/43028)
Supplement: Multimedia Appendix 2 [file resprot_v12i1e43028_app2.pdf]

LSC20-009 Red flags: Verbesserung des Wissens über ernsthafte Pathologien

**Projektnummer:**	LSC20-009
**Projekttitel:**	Red flags: Verbesserung des Wissens über ernsthafte Pathologien
**Hauptantragsteller:**	Jessica Janssen, IMC University of Applied Sciences Krems
**Wissenschaftlicher Leiter:**	Jessica Janssen, IMC University of Applied Sciences Krems
**Projektpartner:**	Christian Keip, IMC University of Applied Sciences Krems<br>Manfred Wieser, Karl Landsteiner University of Health Sciences<br>Florian Rausch, Karl Landsteiner University of Health Sciences<br>Martin Pecherstorfer, Karl Landsteiner University of Health Sciences
**Projektdauer:**	36 Monate
**Fördersumme:**	293.967,53

Begutachtung durch JurorIn 1:

**K1 Qualität des Vorhabens / Qualifikation**	
**Gutachten zu K1:**	

Der Projektantrag "Red flags: Improving knowledge of serious pathologies" beinhaltet die Stärkung des Wissens von Physiotherapeuten/innen über sogenannte "red flags" in der Behandlung von Krankheiten des Bewegungsapparates. Wenn diese "red flags" bekannt bzw. an Fallvignetten trainiert worden sind, können ernsthafte Erkrankungen (z.B. Metastasen und/oder Frakturen) schneller erkannt und die Patienten/innen rascher einer speziellen Diagnostik und Therapie zugeführt werden.

Das Projekt ist gesundheitspolitisch wichtig, der Erkenntnisgewinn ist hoch und eine publikatorische Verwertung erwartbar. Die Niederlande (das Ursprungsland der Antragstellerin) hat im Bereich der Akademisierung des "allied personal" einen weltweit exzellenten Ruf, der mit diesem Antrag wieder bestätigt wird. Der Antrag stärkt den multidisziplinären Ansatz in der Diagnostik und Versorgung muskuloskeletaler Erkrankungen (Physiotherapeuten & Ärzte).

Die Antragstellerin hat einen hervorragenden CV, auch auf ihr akademisches Alter bezogen. Auch der Publication track record - zumindest der angegebenen 10 rezenten Publikationen ist exzellent.

**K2 Arbeitsmöglichkeiten / wissenschaftliches Umfeld:**	
**Gutachten zu K2:**	

Die personelle, institutionellen und räumlichen Voraussetzungen scheinen gegeben zu sein, da apparative Methoden nicht vorgesehen sind.

**K3 Ziele und Arbeitsprogramm:**	
**Gutachten zu K3:**	

Die Arbeitshypothesen sind klar begrenzt und relevant. Der Projektplan sowie die vorgesehen Methoden erscheinen adäquat. Insgesamt scheint das Projekt in der vorgesehenen Zeit umsetzbar.

**K4 Vorschlag zum Umfang der Förderung:**	
**Gutachten zu K4:**	

Nachdem keinerlei Geräte & Laborversuche notwendig sind, beschränkt sich die angesuchte Förderung auf Personalkosten, wobei hier vor allem die Finanzierung einer 75% Stelle (NN1) mit 119,348 Euro dominiert. Inwiefern die restlichen Personalkosten reduziert werden können, sollte bei reduzierten Budget mit der PI besprochen werden.

**Gesamtgutachten:**

Insgesamt halte ich das Projekt für absolut förderungswürdig, da:

1. Qualitätssteigerung: durch das Projekt ist eine Verbesserung der physiotherapeutischen Therapie muskuloskeletaler Erkrankung zu erwarten

2. Akademisierung: durch das Projekt wird der exzellente Ruf "IMC University of Applied Sciences Krems" in diesem Fachbereich gestärkt
3. Publikation: aufgrund des bisherigen publication track record ist eine überdurchschnittlich platzierte Veröffentlichung in einem internationalen Journal zu erwarten

Einschränkung: Ich empfehle die Einbeziehung nationaler oder internationaler orthopädischer Fachgesellschaften (ÖGO, EFORT, ...) , um die Ergebnisse & Maßnahmen auch in einen nationalen bzw. ggf. auch internationalen Kontext bzw. deren Therapiealgorithmen zu setzen.

Begutachtung durch JurorIn 2:

**K1 Qualität des Vorhabens / Qualifikation**	
**Gutachten zu K1:**	

Das antragsstellende Konsortium legt einen Antrag über ein Forschungsvorhaben mit dem Thema der „Red Flags: Verbesserung des Wissens über ernsthafte Pathologien“ vor. Unter Red Flags beim Kreuzschmerz werden im allgemeinen Warnsymtome bezeichnet, die den unspezifischen von einem spezifischen Kreuzschmerz bei einer sorgfältig erhobenen Anamnese und Krankenuntersuchung unterscheidbar machen. Dies ist wichtig bei so häufigen Symptomen wie dem Kreuzschmerz, dem häufig keine schwerwiegenden Pathologien zugrunde liegen. Die Warnsymptome lassen in der Regel schwerwiegende dem Kreuzschmerz zugrundeliegende Krankheiten identifizieren. Diese Warnsymptome sind allgemein anerkannte und beachtete diagnostische Kriterien im Umgang mit dem Kreuzschmerz. Diesbezüglich werden vielfache Literaturangaben gegeben und rezente Arbeiten zitiert.
Die Antragsteller identifizieren einen geringen Bekanntheitsgrad dieser Red Flags und eine Unterqualifikation von Physiotherapeuten in der Beurteilung von Red Flags in der bisherigen Literatur aus nicht ganz rezente Arbeiten aus den Vereinigten Staaten/Deutschland/Schweiz (2004 - 2011). Darauf aufbauend zitieren die Antragsteller eine rezente eigene Arbeit, die aufzeigt, dass unter Allgemeinärzten und Orthopädischen Chirurgen die Meinung vorherrscht, dass die Notwendigkeit einer kompetenten Beurteilung von Red Flags durch Physiotherapeuten notwendig ist um kranke Patienten notwendigerweise einer ärztlichen Behandlung zuzuführen. Eine weitere selbstzitierte Literaturstelle einer randomisierten Pilotstudie unter österreichischen Physiotherapiestudenten zeigt ebenfalls die Notwendigkeit eines zusätzlichen Trainings in der Erkennung von Red Flags auf (W. Lackenbauer 2020).
Die Antragssteller zeigen in rezenter Literatur die allgemeine Belastung der Gesellschaft und steigende Gesundheitskosten durch den Kreuzschmerz auf und betonen die Notwendigkeit zur Erkennung ernster Pathologien im Bereich des muskuloskelettalen Systems. Die gesellschaftliche Überalterung verstärkt laut Antragstellern das zahlmäßige Problem unerkannter oder zu spät erkannter schwerwiegender Pathologien als Gründe für den Kreuzschmerz. Durch dieses Forschungsvorhaben thematisieren und konkretisieren die Antragsteller die Wichtigkeit von Physiotherapeuten in der Unterstützung des Arztes als Gatekeeper in der primären Gesundheitsversorgung. Die Idee der Einbindung nicht ärztlicher Gesundheitsberufe wie z.B. Physiotherapeuten in das Erkennen von Red Flags ist originell und innovativ. Die Erkennung ernster Pathologien durch niedergelassene, selbständig arbeitende Physiotherapeuten und die konsekutive Weiterleitung an Allgemeinärzte, Orthopäden und Internisten wäre ein wichtiger Beitrag zur Verbesserung der Grundversorgung von Patienten in Österreich.
Zur Erstellung des inhaltlichen Backgrounds und der Hinführung zur Forschungsfrage wird zu einem großen Teil bekannte, hochrangige Literatur (Journale mit IF Top 40% der jeweiligen Fachkategorien) verwendet.
Ein Erkenntnisgewinn des Forschungsvorhabens kann in mehrfacher Hinsicht erwartet werden. Zum Ersten ist ein hoher Erkenntnisgewinn für auszubildende Physiotherapeuten zu erwarten. Zum anderen wird ein hoher Erkenntnisgewinn der fächerübergreifend erstellten klinischen Fallvignetten erwartet, die dann zu Ausbildungszwecken den Physiotherapiestudenten vorgelegt werden. Aufgrund der Fächer und Disziplinen übergreifend (Ortho/Interne/Arzt/Physiotherapeut) erstellten und anschließend im Konsensverfahren getesteten/ausgewählten Fallvignetten ist hier von einer höchsten Qualität auszugehen.
Die Entwicklung und Testung von interdisziplinär erstellten Lernunterlagen kann ein Vorbild für viele andere Ausbildungsbereiche in Gesundheitsberufen darstellen. Diese Dimension sollte von den Antragsstellern diskutiert werden.
Die besondere Bedeutung des gegenständlichen Antrags steckt aber in sekundären gesundheitsökonomischen, gesellschaftspolitischen und sozioökonomischen Potentialen. Das strukturierte Lernen von Red Flags durch klinische Fallvignetten bei Physiotherapeuten kann bei erfolgreicher Abwicklung des Projektes auch auf andere Gesundheitsberufe wie diplomierte

KrankenpflegerInnen, OsteopathInnen, MasseurInnen und SportwissenschaftlerInnen erweitert werden. Die breite Kenntnis von Fallvignetten und schweren Pathologien kann auch zu einem höheren Gesundheitsbewusstsein in der gesamten Gesellschaft führen. Es wäre denkbar, dass die Früherkennung von schweren Pathologien zu rechtzeitig eingeleiteten adäquaten Therapien und zur Reduktion von stationären Krankenhausaufenthalten und Kosten im Gesundheitssystem führen könnte. Auch wenn das Vorhaben im Wesentlichen die Erstellung hochqualitativer Lehrunterlagen für Physiotherapeuten und den Prozess des Lernens dieser Inhalte zum Ziel hat, so sind die genannten gesamtheitlicheren Implikationen wohl ableitbar.

Die Antragsteller könnten noch mehr auf die Rolle des Prinzips des Gatekeepings speziell im Österreichischen Gesundheitswesen eingehen. Welche Verbesserungen sind durch die Aufwertung der Physiotherapeuten konkret zu erwarten? Wieviele Patienten könnten frühzeitig erkannt werden? Gibt es hier rechnerische Überlegungen Aus dem Bereich der Public Health? Gibt es hier Beispiele aus anderen Ländern wie sich eine solche Maßnahme/Verbessertes Erkennen ausgewirkt haben, Niederlande z.B.? Diese potentiellen Verbesserungen der primären Gesundheitsversorgung werden im Antrag nur oberflächlich diskutiert. Da diese Überlegungen den Antrag aber massiv stärken sollten die oben gestellten Fragen beantwortet werden.

**K2 Arbeitsmöglichkeiten / wissenschaftliches Umfeld:**	
**Gutachten zu K2:**	

Im Rahmen des Forschungsvorhabens werden in einem hochwissenschaftlichen Umfeld 4 Vertragspositionen (N.N.1-4) geschaffen für die ein Erkenntnisgewinn vorhersehbar ist. Von entsprechenden räumlichen Arbeitsmöglichkeiten ist an den beteiligten Institutionen auszugehen, wenngleich in den einzelnen Arbeitspaketen eine genauere Beschreibung der räumlichen Infrastruktur fehlt. Im Arbeitspaket 2.0 wird ein Survey im Rahmen eines online-Formates durchgeführt. Die Entwicklung und Validierung der klinischen Vignetten wird im Rahmen von regelmäßigen Meetings in einem speziellen Konsensprotokoll kreiert werden. Der genaue Ort dieser Meetings wird nicht bekanntgegeben. Als Arbeitsgruppe im Rahmen der Erarbeitung der klinischen Fallvignetten im iterativen Design-Prozess wird eine Gruppe von 9-15 Physiotherapeuten, Lehrende und medizinische Experten angegeben, die im Rahmen der geplanten 3 oder 4 Meetings zur Entwicklung von 20 neuen Fallvignetten beitragen sollen. Die Auswahl und Anforderungen der Physiotherapeuten dieser Arbeitsgruppe ist nicht näher spezifiziert. Ob es außer den Meetings noch sonstigen räumlichem Bedarf geben wird, ist nicht festgelegt, aber anzunehmen. Es wird nicht darauf eingegangen inwiefern virtuelle alternativen zu analogen Meetings stattfinden könnten. Ob das Projekt auch unter verschärften Versammlungseinschränkungen (z.B. COVID-19 Maßnahmen) zu realisieren ist wird nicht dargelegt. Desgleichen wird auch nicht dargelegt an welcher Örtlichkeit die spezielle Zusammenarbeit des medizinischen Masterstudenten und Physiotherapeuten zur Analyse und Vergleich der klinischen Beispiele der Fallvignetten und dem Abgleich mit dem International Framework for Red Flags erfolgen wird und ob Gerätschaften (IT-Programme) und räumliche Strukturen für einen reibungslosen Ablauf gewährleistet sein werden.

Der Interventionspart der Studie und das Unterrichten der Red Flags wird an 30 Physiotherapeuten am IMC FH Krems stattfinden.

**K3 Ziele und Arbeitsprogramm:**	
**Gutachten zu K3:**	

Die Ziele und das wissenschaftliche Arbeitsprogramm werden unter Punkt 3.3 bzw. Punkt 3.7 des Antrags dargelegt.

Im Arbeitspaket 2.1. soll das aktuelle Wissen über Red Flags von österreichischen Physiotherapeuten anhand bereits validierter und gebrauchter Vignetten aus dem Jahr 2006 in einem Survey evaluiert werden. Hier besteht ein Informationsdefizit hinsichtlich der Anzahl und des Erfahrungsstandes der einzelnen einzuschließenden Physiotherapeuten. Es gibt keine genaue Angabe über die Auswahlkriterien der Physiotherapeuten zu diesem Survey.

Das Arbeitspaket 2.2 besteht aus einem Methodenmix zur Entwicklung neuer klinischer Fallvignetten in einer multidisziplinären Arbeitsgemeinschaft aus Ärzten und Physiotherapeuten in einem gemeinsamen iterativen Designprozess. Diese interdisziplinäre Herangehensweise in der Erstellung, aber auch in der Validierung der Fallvignetten beschreibt den Erkenntnisprozess des Forschungsvorhabens. Zur Einigung/Auswahl der Vignetten wird das Verfahren der HAS Konsensus Methode verwendet, die ausgewählten Vignetten werden schließlich im joint effort einer MedizinMasterstudentIn und einer PhysiotherapeutIn mit dem international Framework for Red Flags abgeglichen. Vorzuge des HAS Konsensus sollten diskutiert werden und warum gerade diese Form des Konsenses gesucht wird.

Das Arbeitspakets 2.3 beschreibt eine qualitative Interventionsstudie mittels strukturierter Interviews an 30 teilnehmenden Physiotherapeuten der IMC FH Krems. Dieses Arbeitspaket ist hinsichtlich der zu erwartenden Ergebnisse unklar formuliert. Es besteht Unklarheit, ob hier ein vermehrtes Verständnis der Physiotherapeuten durch die neuen Fallvignetten im Rahmen der strukturierten Interviews abgefragt oder ein Feedback betreffend den Lernprozess erfasst werden soll. Hier sollten die Ziele klarer definiert werden.

Abgesehen vom AP 2.3. erscheinen die Arbeitspakete und Ziele klar und eingegrenzt durchgängig definiert. Methodisch besteht ein Mixed-Methoden-Ansatz. Dabei kommen eine Umfrage, eine Konsensusmethode und eine qualitative Studie mit strukturierten Interview, geschlossenen und offenen Fragen zur Anwendung.

Die Ziele der Arbeitspakete und Sub-Tasks erscheinen in den vorgegebenen Zeiträumen durchführbar. Die Dauer des Arbeitspakets 2.3 über 1 ½ erscheint lange. Diesbezüglich wäre eine genauere Strukturierung des Ablaufes notwendig. Strukturierte Interviews und qualitative Messmethoden erfordern viel Zeit. Dennoch erscheint ein Lernmodul mit 20 klinischen Vignetten mit 30 Physiotherapeuten für 1 ½ Jahre zeitlich überdimensioniert. Empfehle hier die Klärung, ob serielle Mehrfachinterviews aller Physiotherapeuten erfolgt eine einmalige Befragung? Werden hier nur subjektive Eindrücke der Physiotherapeuten über das neugewonnene Wissen oder auch objektiv gebessertes neues Wissen abgefragt?

**K4 Vorschlag zum Umfang der Förderung:**	
**Gutachten zu K4:**	

Ein Großteil der Kosten sind Personalaufstellung, insbesondere des Masterstudenten, der über 3 Jahre über das Projekt finanziert werden soll. Hinzu kommt die bezahlte Expertise des internationalen Experten. Der Umfang der Förderung des Personalbedarfs erscheint gerechtfertigt. Auffällig ist der hohe In-kind Beitrag der von den Projektbeteiligten eingebracht wird, was die Projektkosten relativiert.

**Gesamtgutachten:**

Hinsichtlich der im Punkt 2.0 Ziele, der Richtlinien für die Förderung wissenschaftlicher Projekte ist zu dem gegenständlichen Antrag folgendes zu sagen:

Stärkung vorhandener Forschungskompetenz auf dem Feld der Live Science

Das zur Begutachtung vorliegende Projekt wird an zwei niederösterreichischen akademischen Institutionen, nämlich der IMC-Fachhochschule Krems sowie der Karl Landsteiner Privatuniversität für Gesundheitswissenschaften durchgeführt. Die Projektleitung (JJ) zeichnet sich durch eine hohe Lehr- und Wissenschaftskompetenz im dargestellten Fachbereich der Physiotherapie aus. Durch ihre im In- und Ausland über viele Jahre ihres akademischen Curriculums gewonnene klinische und akademische Expertise wird dieses Feld an diesen Wissenschaftsstandorten gestärkt. Durch die Vernetzung von Orthopädie/Trauma, Innere Medizin/Onkologie und Physiotherapie besteht potentiell die interdisziplinäre Vermehrung von Forschungskompetenz. Die Beteiligung eines international anerkannten Experten JS wird die vorhandene Forschungskompetenz zusätzlich stärken. Außerdem werden 4 Mitarbeiter (MasterstudentIn und PhysiotherapeutIn als auch 2 weitere nicht näher spezifizierten Projektangestellte) von diversen angeleiteten Arbeitsschritten und Anwendung diverser Methoden qualitativer und quantitativer Messmethoden profitieren wodurch die lokale Forschungskompetenz im Feld der Life-Science steigt.

Stärkere Vernetzung Niederösterreichischer Forschungseinrichtungen

Durch den interdisziplinären Ansatz des vorliegenden Projektes werden medizinischen Fachrichtungen, verschiedene Gesundheitsberufe und unterschiedlicher akademischer Einrichtungen in Niederösterreich miteinander vernetzt.

Beitrag zur Verwirklichung von mittelfristigen Nutzen und Verwertungspotenzialen

Das Projekt zielt auf eine verbesserte Ausbildung von Physiotherapeuten hinsichtlich der Fähigkeiten eines verbesserten Erkennens von Pathologien im Bereich des muskuloskelettalen Apparates ab. Es ist möglich, aber nicht Gegenstand dieses Forschungsvorhabens, dass sich aus einer generellen Bewusstmachung schwerwiegender Pathologien für Vertreter diverser Gesundheitsberufe, eine Verbesserung in der primären Patientenversorgung ableiten lässt. Die Früherkennung inzipienter schwerer Pathologien im Bereich der Wirbelsäule könnte allerhöchste Relevanz durch eine Frühanbindung an eine ärztliche Behandlung und möglichweise auch Vermeidung langer und Kostenintensiver Krankenhausaufenthalte und – behandlungen in sich bergen. Das gegenständliche Forschungsvorhaben hat somit potentiell einen großen mittelfristigen Nutzen wenn eine Weiterverbreitung des neugewonnenen Wissens erfolgt. Die Weiterverbreitung im Sinne der Erschaffung einer APP für den täglichen Gebrauch für Physiotherapeuten ist in einem weiteren Förderantrag (BRIDGE FFG) geplant, jedoch nicht Gegenstand dieses Antrags. Auch ist im gegenständlichen Forschungsvorhaben keine Sicherstellung einer dauerhaften Verbesserung in der Ausbildung von Physiotherapeuten dezidiert festgehalten. Eine dauerhafte Implementierung der entwickelten Inhalte wäre wünschenswert und sollte durch die Antragsteller veranlasst werden.

Insgesamt halte ich das Projekt für ein exzellentes Forschungsvorhaben mit nachvollziehbarem Finanzierungsplan. Unbedingt sind für mich allerdings die zwei, von mir in der Begutachtung erwähnten, wesentliche Ergänzungen nachzureichen. Die Antragsteller sollten

die Dimension einer Verbesserung der Primärversorgunug, wie von mir in der Begutachtung erwähnt, abschätzen/skizzieren. Weiters müssen die Ziele des 3. Arbeitspaketes, wie in der Begutachtung dargestellt, klarer formuliert werden.
